# Supplementary material for: Transitions in intensive care: Investigating critical slowing down post extubation
Source: PLoS One. 2025 Jan 24;20(1):e0317211. doi: 10.1371/journal.pone.0317211 (PMC11760018; doi:10.1371/journal.pone.0317211)
Supplement: S1 File — In this supplementary we discuss the reasons for missing records in the data and present the results of how the mean characteristics of the data vary between Cohorts 1 and 2. (PDF) [file pone.0317211.s001.pdf]

Supplementary 1: Data characteristics

Lucinda Khalil[1], Sandip V George[2,3], Katherine L. Brown[4], Samiran Ray[5], and Simon Arridge[2]

[1]Department of Mathematics, Imperial College London, London, UK
[2]Department of Computer Science, University College London, London, UK
[3]Department of Physics, University of Aberdeen, Aberdeen, UK
[4]Cardiac Intensive Care Unit, Great Ormond Street Hospital For Children NHS Foundation Trust, London, UK
[5]Paediatric Intensive Care Unit, Great Ormond Street Hospital For Children NHS Foundation Trust, London, UK

In this section we present the reasons for missing values and the differences in the mean characteristics between the datasets used in the analysis.

1 Reasons for data loss

Three main reasons are identified for data loss. The first occurs due to the condition imposed on the duration of extubation (listed as R1 in Table 1). The second occurs when the total number of values in the time series is less than 1.5 times the extubation duration, after pre-processing (listed as R2). The third was loss of data during the calculation of early warning signals

The mean characteristics are described in Table 2. For the heart rate and respiratory rate, where age related reference values are available, an age normalized z-score is compared instead of the mean values[?]. Welch t-tests that do not assume equal variance between the distributions are used to compare the two cohorts.

While the ages do not significantly differ between the two cohorts due to stratification, both age normalized measures show significant differences. The arterial blood pressure where age related reference values could not be used for normalization too showed a significant difference between the two groups.

	Coh1				Coh2			
	N	R1	R2	R3	N	R1	R2	R3
HR	183	52	2	28	1624	0	71	78
RR	183	52	27	3	1624	0	324	60
ABP	168	44	2	63	1487	0	406	48

Table 1: Number of missing records for each file. N: Total number of records; R1: Extubation time<120 minutes; R2: Total number of points after pre-processing < 1440; R3: Data loss during calculation of EWS.

Measure	μ_{Coh1} (SD)	μ_{Coh2} (SD)	z-value	p-value
Age	3.249 (4.767)	3.211 (4.947)	-0.075	0.94
HR (Age normalized)	**0.010 (.015)**	**.004(.015)**	**4.322**	**<.001**
RR (Age normalized)	**-0.049 (.006)**	**-0.051 (.006)**	**3.090**	**.003**
ABP	**76.704 (15.895)**	**71.684 (11.892)**	**2.575**	**0.012**

Table 2: Differences in the average characteristics between cohorts 1 and 2 When a predictor was significant in the model ($p < .05$), the corresponding row is shown in bold.
HR: Heart rate; RR:Respiratory rate; ABP: Arterial blood pressure.
