# Supplementary material for: Transitions in intensive care: Investigating critical slowing down post extubation
Source: PLoS One. 2025 Jan 24;20(1):e0317211. doi: 10.1371/journal.pone.0317211 (PMC11760018; doi:10.1371/journal.pone.0317211)
Supplement: S2 File — In this supplementary we explore how the proportions of records that show critical slowing down in cohorts 3 and 4 vary from cohort 2. (PDF) [file pone.0317211.s002.pdf]

Supplementary 2 : Comparisons of cohorts 3 and 4 with control

Lucinda Khalil[1], Sandip V George[2,3], Katherine L. Brown[4], Samiran Ray[5], and Simon Arridge[2]

[1]Department of Mathematics, Imperial College London, London, UK
[2]Department of Computer Science, University College London, London, UK
[3]Department of Physics, University of Aberdeen, Aberdeen, UK
[4]Cardiac Intensive Care Unit, Great Ormond Street Hospital For Children NHS Foundation Trust, London, UK
[5]Paediatric Intensive Care Unit, Great Ormond Street Hospital For Children NHS Foundation Trust, London, UK

1 Comparison of Cohort 3 With Control Cohort 2

The comparison conducted between cohort 1 and cohort 2 (control group) was also carried out between cohort 3 and cohort 2. Table 1 shows the proportions of significant results for the two cohorts when considered as a whole, as well as when split by ICU types. We notice that the proportions of critical slowing down observed in cohort 3 are remarkably close to the control group, cohort 2. No significant differences were observed in the proportions of datasets showing significant increases in any of the quantifiers considered, between cohorts 3 and 2. We also observe no differences when the data is split by ICU ward type.

2 Comparison of Cohort 4 With Control Cohort 2

The same process was also carried out on cohort 4 which is made up of patients who died post re-intubation. Results for the analysis on cohort 4 are presented in Table 2. On this occasion, when the entire cohort is considered, 1 of the 6 tests show significant results. More specifically, the autocorrelation of the mean blood pressure provided an indication of critical slowing down. The PICU ward showed significant results in 2 of the 6 cases considered, namely the autocorrelation and variance of the mean blood pressure. The confusion matrices, positive predictive values, negative predictive values, sensitivities and specificities for Cohorts 3 and 4 are presented in Table 3. Due to the small sample size of cohort 4, any conclusions drawn from the analysis must be treated with caution.

	Variance, σ^2						Autocorrelation, r_1					
	HR		RR		ABP		HR		RR		ABP	
	C3	C2	C3	C2	C3	C2	C3	C2	C3	C2	C3	C2
CICU	0.251	0.251	0.312	0.315	0.189	0.187	0.180	0.179	0.106	0.108	0.182	0.183
NICU	0.323	0.330	0.306	0.304	0.269	0.261	0.227	0.232	0.116	0.110	0.269	0.305
PICU	0.225	0.221	0.242	0.236	0.172	0.170	0.124	0.119	0.169	0.180	0.129	0.134
Whole	0.254	0.254	0.293	0.294	0.188	0.186	0.172	0.171	0.123	0.126	0.175	0.178

Table 1: The proportions of significant Mann-Kendall hypothesis tests conducted on cohort 3. The first 3 rows show these proportions if only one ICU ward is considered at a time. The pairs of proportions which are significantly different between cohort 3 and 2 are highlighted.

	Variance, σ^2						Autocorrelation, r_1					
	HR		RR		ABP		HR		RR		ABP	
	C4	C2	C4	C2	C4	C2	C4	C2	C4	C2	C4	C2
CICU	0.0	0.251	0.25	0.315	0.25	0.187	0.0	0.179	0.0	0.108	0.25	0.183
NICU	0.5	0.329	0.000	0.305	0.000	0.261	0.5	0.232	0.000	0.110	0.000	0.304
PICU	0.333	0.220	0.167	0.236	**0.429**	**0.170**	0.250	0.119	0.167	0.180	**0.571**	**0.134**
Whole	0.278	0.254	0.176	0.294	0.333	0.185	0.222	0.171	0.117	0.126	**0.417**	**0.178**

Table 2: The proportions of significant Mann-Kendall hypothesis tests conducted on cohort 4. The first 3 rows show these proportions if only one ICU ward is considered at a time. The pairs of proportions which are significantly different between cohort 4 and 2 are highlighted.

**COHORT 3**	Measure	TP	FP	FN	TN	PPV	NPV	Sensitivity	Specificity
Variance, σ^2	HR	19	374	54	1101	0.070	0.949	0.302	0.746
	RR	15	365	41	875	0.039	0.943	0.268	0.706
	ABP	11	192	34	841	0.054	0.972	0.244	0.814
Autocorrelation, r_1	HR	14	253	59	1222	0.036	0.954	0.192	0.828
	RR	4	156	52	1084	0.025	0.954	0.071	0.874
	ABP	3	184	40	849	0.026	0.955	0.111	0.822
**COHORT 4**	Measure	TP	FP	FN	TN	PPV	NPV	Sensitivity	Specificity
Variance, σ^2	HR	5	374	13	1101	0.0132	0.988	0.277	0.746
	RR	3	365	14	875	0.008	0.984	0.176	0.706
	ABP	4	192	8	841	0.020	0.990	0.333	0.814
Autocorrelation, r_1	HR	4	253	14	1222	0.016	0.989	0.222	0.828
	RR	2	156	15	1084	0.013	0.986	0.118	0.874
	ABP	5	184	7	849	0.026	0.992	0.417	0.822

Table 3: Confusion values, PPV, NPV, sensitivity and specificity of the model for cohorts 3 and 4. HR: heart rate (beats per minute/BPM), RR: respiration rate (breaths per minute/BrPM), ABP: average blood flow (millimeters of Mercury/mmHg), TP: true positive, FP: false positive, FN: false negative, TN: true negative, PPV: positive predictive value, NPV: negative predictive value
