# Supplementary material for: Transitions in intensive care: Investigating critical slowing down post extubation
Source: PLoS One. 2025 Jan 24;20(1):e0317211. doi: 10.1371/journal.pone.0317211 (PMC11760018; doi:10.1371/journal.pone.0317211)
Supplement: S3 File — In this supplementary we explore how the results vary when using smaller window sizes of 15 and 30 minutes. (PDF) [file pone.0317211.s003.pdf]

Supplementary 3: Variation with smaller window sizes

Lucinda Khalil[1], Sandip V George[2,3], Katherine L. Brown[4], Samiran Ray[5], and Simon Arridge[2]

[1]Department of Mathematics, Imperial College London, London, UK
[2]Department of Computer Science, University College London, London, UK
[3]Department of Physics, University of Aberdeen, Aberdeen, UK
[4]Cardiac Intensive Care Unit, Great Ormond Street Hospital For Children NHS Foundation Trust, London, UK
[5]Paediatric Intensive Care Unit, Great Ormond Street Hospital For Children NHS Foundation Trust, London, UK

In this section we consider evidence of critical slowing down in the data sets when using smaller window sizes. We use window sizes of 30 and 15 minutes and repeat the analyses described in Section 2 in the main text. The smaller window sizes used can pick up variations at a smaller time scale. Unlike the main analysis, we only compare the variations for cohorts 1 and 2.

1 30 minute windows

We present the proportion of significant tests for cohorts 1 and 2, when using 30 minute windows in Table 1. Overall, we see an increase in the number of false positives throughout the dataset, with no quantifier showing significant differences when the data is considered as a whole, or when split by ICU wards.

2 15 minute windows

Similar analyses was conducted using 15 minute windows, with results presented in Table 2. The autocorrelation of the heart rate shows a significant difference between the proportions of cases showing an increase over time. This quantifier also shows a significant difference between the two cohorts when only the CICU ward is considered.

	Variance, σ^2						Autocorrelation, r_1					
	HR		RR		ABP		HR		RR		ABP	
	C1	C2	C1	C2	C1	C2	C1	C2	C1	C2	C1	C2
CICU	0.174	0.257	0.282	0.317	0.179	0.221	0.239	0.175	0.128	0.105	0.179	0.198
NICU	0.412	0.285	0.071	0.305	0.500	0.273	0.294	0.258	0.214	0.096	0.000	0.136
PICU	0.189	0.252	0.233	0.223	0.208	0.152	0.189	0.151	0.163	0.165	0.250	0.138
Whole	0.215	0.260	0.229	0.292	0.209	0.211	0.224	0.180	0.156	0.119	0.194	0.186

Table 1: The proportions of significant Mann-Kendall hypothesis tests conducted on cohort 1 when using 30 minute windows. The first 3 rows show these proportions if only one ICU ward is considered at a time. The pairs of proportions which are significantly different in the expected direction between cohort 1 and 2 are highlighted.

	Variance, σ^2						Autocorrelation, r_1					
	HR		RR		ABP		HR		RR		ABP	
	C1	C2	C1	C2	C1	C2	C1	C2	C1	C2	C1	C2
CICU	0.132	0.239	0.170	0.319	0.204	0.208	**0.245**	**0.156**	0.106	0.094	0.114	0.188
NICU	0.368	0.305	0.063	0.321	0.500	0.185	0.263	0.262	0.188	0.107	0.000	0.148
PICU	0.367	0.267	0.286	0.176	0.313	0.300	0.200	0.200	0.071	0.176	0.187	0.200
Whole	0.215	0.246	0.153	0.294	0.197	0.198	**0.238**	**0.166**	0.135	0.110	0.145	0.174

Table 2: The proportions of significant Mann-Kendall hypothesis tests conducted on cohort 1 when using 15 minute windows. The first 3 rows show these proportions if only one ICU ward is considered at a time. The pairs of proportions which are significantly different in the expected direction between cohort 1 and 2 are highlighted.
