# Supplementary material for: Transitions in intensive care: Investigating critical slowing down post extubation
Source: PLoS One. 2025 Jan 24;20(1):e0317211. doi: 10.1371/journal.pone.0317211 (PMC11760018; doi:10.1371/journal.pone.0317211)
Supplement: S4 File — In this supplementary we explore whether critical slowing down can be detected in oxygen saturation data, leading up to extubation failure. (PDF) [file pone.0317211.s004.pdf]

Supplementary 4: Critical slowing down in Oxygen saturation data

Lucinda Khalil[1], Sandip V George[2,3], Katherine L. Brown[4], Samiran Ray[5], and Simon Arridge[2]

[1]Department of Mathematics, Imperial College London, London, UK
[2]Department of Computer Science, University College London, London, UK
[3]Department of Physics, University of Aberdeen, Aberdeen, UK
[4]Cardiac Intensive Care Unit, Great Ormond Street Hospital For Children NHS Foundation Trust, London, UK
[5]Paediatric Intensive Care Unit, Great Ormond Street Hospital For Children NHS Foundation Trust, London, UK

In this section we consider evidence for critical slowing down in the oxygen saturation data from cohorts 1 and 2. As before, we use one hour long windows to conduct the analysis. The proportions of significant increases observed in cohorts 1 and 2 for the autocorrelation and variance are shown in Table 1. No significant differences were observed in any of the cases when the cohorts were taken as a whole, or when they were split by ICU type. In Table 2 we show the differences between the mean correlation coefficient observed in the two cohorts. In this case, we see significant differences between the two cohorts for both the autocorrelation and variance, when the data is taken as a whole. When split by ICU types, the PICU shows significant differences for both measures, whereas the CICU shows significant differences for the autocorrelation.

References

	SpO_2			
	Variance, σ^2		Autocorrelation, r_1	
	C1	C2	C1	C2
CICU	0.410	0.293	0.231	0.185
NICU	0.333	0.223	0.267	0.215
PICU	0.267	0.256	0.267	0.203
Whole	0.333	0.275	0.253	0.193

Table 1: The proportions of significant Mann-Kendall hypothesis tests conducted on the ocygen saturation data from cohort 1, when using 60 minute windows. The first 3 rows show these proportions if only one ICU ward is considered at a time. The pairs of proportions which are significantly different in the expected direction between cohort 1 and 2 are highlighted.

	SpO_2					
	Variance, σ^2			Autocorrelation, r_1		
	μ_1	μ_2	p	μ_1	μ_2	p
PICU	**.143 (45)**	**-0.004 (246)**	**0.005**	**0.098 (45)**	**-0.023 (246)**	**0.031**
CICU	0.135 (39)	0.049 (628)	0.255	**0.081 (39)**	**-0.036 (628)**	**0.031**
NICU	0.155 (15)	0.042 (121)	0.337	0.051 (15)	0.005 (121)	0.694
Whole	**0.142 (99)**	**0.03 (995)**	**0.009**	**0.084 (99)**	**-0.028 (995)**	**0.002**

Table 2: Means and sample sizes of Kendall-Tau correlation coefficients of cohort 3 and cohort 2, as well as p-values when the Welch's T-Test is conducted. Statistically significant results are highlighted. Also shows values when grouped by ICU ward type.
