# Supplementary material for: Transitions in intensive care: Investigating critical slowing down post extubation
Source: PLoS One. 2025 Jan 24;20(1):e0317211. doi: 10.1371/journal.pone.0317211 (PMC11760018; doi:10.1371/journal.pone.0317211)
Supplement: Attachment — Submitted filename: SVG_ResponseToReferee.pdf [file pone.0317211.s005.pdf]

Reviewer #2: The authors have significantly revised their manuscript. They highlight that CSD has evidence but low sensitivity and the prevalence of false positives limits utility in this pediatric intensive care unit population. This is a negative result.

While negative results can still be published, the potential benefits should not be oversold. The tone overall should be reduced. I agree with the level of the statement: "While immediate clinical utility is limited, the work marks an important first step towards using dynamical systems theory to understand the dynamics of signals measured at the bedside during intensive care." The rest of the text should be tamed down to match this level.

We agree with the reviewer that use of CSD alone for clinical detection of extubation failure is limited. Hence we remove the following sentence from the abstract.

'Our results suggest that the autocorrelation of the heart rate acts as an early warning signal (EWS) for failure post extubation.'

However we point out that the exploratory analyses carried out in Section 3.4, show that the information contained in the variables that capture CSD is complementary to the information contained in commonly used variables such as the mean and standard deviation of the data. Hence, while CSD alone may not be a viable predictor of extubation failure, it shows potential in enhancing the predictability of existing models. This has now been highlighted in the abstract, as
'Moreover, incorporating these magnitudes significantly improved the fit of a logistic regression model when compared to a model that solely used the mean and standard deviation of the vital signs.'

Table 1 is insufficiently detailed.

Table 1 has now been expanded to include details of the broad diagnostic categories patients fell into at the time of admission to the various ICU wards (PICU/NICU and CICU).

The text on tracheostomies is an unnecessarily dramatic addition. Please revise the text to reflect more common practice.

The text on tracheostomies was included in response to a query from a previous reviewer and was written by a pediatric critical care consultant. To the best of our knowledge this reflects common practice. However, we have now condensed the text from the previous version in response to the reviewer comments.

Discussing previous attempts to predict extubation outcomes doesn't need to come from the same dataset; what techniques have been attempted in other datasets?

The introduction has now been expanded to mention techniques from statistics and machine learning that have been used to predict extubation failure.

‘A number of attempts have been made at predicting extubation failure and re-intubation using statistical and machine learning methods. These include logistic regression, support vector machine, random forest, gradient boosting methods and neural networks. These methods showed varying degrees of success (AUC between .7 and .9) depending on the specifics of the algorithm and the dataset that was used for prediction\cite{mueller2013can, miu2014predictors, hsieh2018artificial, zhao2021development, guzatti2022predictive}. Most of these methods used a feature set consisting of measurements such as the length of ICU stay, pre-existing comorbidities, Glasgow Coma Scale score and averages of different vital signs\cite{torrini2021prediction}. These approaches largely ignore the nonlinear dynamics of measured vital signs, particularly whether patterns in their history could inform extubation outcome.’

Displaying data on the patient could involve graphs and other methods that do not break privacy/HIPAA.

We understand that this remark is in response to a question by referee 1 ‘If patient FR3426 is going to be mentioned, data for this patient should be shown in the supplementary materials.’ We agree with the reviewer’s comment, and the graph for the HR, RR and ABP for patient FR3426 is shown in Figure 4.

Please fix spelling and grammar throughout

Spelling and grammar was rechecked and corrected as appropriate in different parts of the manuscript
