# Supplementary material for: Transitions in intensive care: Investigating critical slowing down post extubation
Source: PLoS One. 2025 Jan 24;20(1):e0317211. doi: 10.1371/journal.pone.0317211 (PMC11760018; doi:10.1371/journal.pone.0317211)
Supplement: Attachment — Submitted filename: ResponseToReferee_PONE-D-24-07937-2.pdf [file pone.0317211.s006.pdf]

PONE-D-24-07937: Response to Referees

We thank the editor and the referees for their questions and comments. Modifications made in response to these have improved the manuscript considerably, and we hope that the referees and the editor are satisfied with the revisions made. Queries are marked in bold and the answers are given in italics.

Editor:
enhancing the method section with a graph to illustrate the step-by-step data collection process can significantly boost clarity. Moreover, further explanation of both the statistical and clinical methodologies and their correlation is needed.

Thank you for these comments. In line with these suggestions, a new figure (Figure 1) has been included in the revised version of the manuscript, detailing the steps of data collection and segregation into data cohorts. In addition, we have expanded the introduction by incorporating references to previous work, which motivates how the statistical techniques used in this manuscript can be used to answer questions in the context of medicine in general and critical care in particular. These are elaborated further below.

Reviewer #1: Thanks for the interesting idea and elaborative work.
I believe your methodology needs more explanation and correlation with the clinical basis.
I believe that the statistical methodology needs more explanation in correlation with the medical part.

Thank you for this comment. In line with this suggestion, we have now expanded the introduction to motivate how the statistical techniques that have been used in this study are relevant to medicine and critical care. In particular we have added references to previous literature which suggests that extubation may be accompanied by slowing of recovery, as expected in critical slowing down. The modifications made to the introduction are mentioned below.

‘In the context of medicine, slowing down of recovery has been suggested as a sign of proximity to a tipping point [13]. This has been observed in contexts such as blood pressure regulation, where slow recovery from changes in blood pressure has been linked to increased risk of mortality or of ischemic stroke [13–16].‘

‘It has been shown previously that slow recovery to baseline levels of minute ventilation following successful spontaneous breathing trials is a risk factor for failure of extubation [30, 31]. Such slow recovery to baseline levels is in line with CSD prior to critical transitions in nonlinear dynamical systems [13].’

Reviewer #2: Dear authors, many thanks for your valuable work. Could you please, add a graph to illustrate the method section clearly? I think it will support the method section. It will focus on the process of data collection step by step.

We thank the referee for their suggestion. In line with this comment, we have now added a figure (Figure 1) in the revised manuscript to illustrate the data collection and segregation into cohorts.

Reviewer #3: This study is interesting for the readers.
It is scientifically sound and contains sufficient interest and originality to merit publication.
This paper is an important contribution and I recommend that it be accepted for publication.

We thank the referee for their kind comments.
