# Supplementary material for: Transitions in intensive care: Investigating critical slowing down post extubation
Source: PLoS One. 2025 Jan 24;20(1):e0317211. doi: 10.1371/journal.pone.0317211 (PMC11760018; doi:10.1371/journal.pone.0317211)
Supplement: Attachment — Submitted filename: ResponseToReferee_PONE-D-24-07937R1-SVG.pdf [file pone.0317211.s007.pdf]

Response letter: PONE-D-24-07937R1

We thank the editor for their detailed review of the manuscript which has helped improve it significantly. We have addressed specific concerns raised by the editor in the following paragraphs. The queries have been shown in bold, whereas the relevant text in the main manuscript is shown in italics. Specific text added in response to the queries have been additionally highlighted in yellow.

The revised manuscript has improved significantly with the addition of the new figure and expanded introduction. However, further clarification and expansion of the methods, results and discussion sections would help strengthen the manuscript and make it suitable for publication.This is to provide more context, justification and discussion around the findings to give readers a complete picture of the study

We thank the editor for their kind comments on the revisions made. We believe that the changes made in response to the comments below have improved the manuscript considerably and makes it more suitable for publication.

Introduction

Q1. The introduction could be further expanded to provide more clinical context and motivation for using critical slowing down (CSD) to study extubation failure. Specifically: Provide statistics on extubation failure rates and associated complications to highlight the clinical importance of the problem

We thank the editor for these comments. The introduction has been expanded considerably to include more text providing motivation for using CSD to study extubation failure, and the statistics of extubation failure and complications associated with it. Reported extubation failure rates vary between 5-15% of cases. This failure can result in poorer outcomes, including higher mortality and increased disease severity. On the other hand prolonged mechanical ventilation is associated with ventilator-associated pneumonia and muscle atrophy. Hence, detecting extubation failure quickly is critical, as delayed re-intubation correlates with higher mortality. Critical slowing down (CSD) is associated with sudden transitions in dynamical systems, and has been observed in multiple real world contexts. However, no investigation has been conducted so far to explore CSD prior to extubation failure. Further motivation for why CSD could work in this context is provided in response to Q2 and Q3.

Prolonged mechanical ventilation is associated with a large number of complications, including risk of ventilator-associated pneumonia, muscle atrophy, as well as other physiological and psychological complications[28], while premature extubation is associated with failure as described below[29].

In 5-15% of cases, extubation can be unsuccessful and the breathing tube may need to be reintroduced, resulting in what is called an 'extubation failure'[30,31]. This can be relatively immediate, or may become apparent over a period of hours. Extubation failure is associated with generally poorer outcomes including

higher mortality and increased disease severity[32]. Detection of this ’extubation failure’ may be useful to prepare for re-intubation, reducing the risk from an emergency procedure. Even post extubation, it is important to predict failure quickly, since a longer time to reintubation is associated with higher mortality [33].

Q2. Discuss in more detail how slowing of recovery dynamics has been observed in other medical contexts as a sign of proximity to a critical transition, to strengthen the rationale for investigating CSD in extubation failure.

The introduction has been expanded with examples of how slowing down of recovery has been observed in other medical contexts, such as blood pressure regulation, seizure prediction and in psychiatric disorders. In all these cases, CSD was detected close to critical transitions.

This has been observed in contexts such as blood pressure regulation, where slow recovery from changes in blood pressure has been linked to increased risk of mortality or of ischemic stroke [13–16] and in seizure prediction, where CSD has been reported in computational, in-vitro [17, 18], as well as in real data [19]. Critical slowing down is also thought to precede transitions in psychiatric disorders, including bipolar disorder and depression [20–22].

Q3. Mention any prior work that has looked at dynamics of vital signs around extubation, to position this study in the context of existing literature.

Despite the large number of studies on understanding and predicting extubation failure, investigations on the dynamics of vital signs near extubation failure have been fewer. We have now expanded the introduction with details on a few relevant studies which showed how dynamics of vital signs relate to extubation outcome.

A smaller number of studies have investigated extubation failure by studying the dynamics of vital signs time series. White et. al. demonstrated the association between lower interbreath interval complexity and extubation failure[40]. Seely et. al. showed that the heart and respiratory rate variability recorded prior to extubation was significantly associated with extubation outcome [41]. Keim-Malpass et. al. investigated how adding variables based on the dynamics of vital signs resulted in a significant increase in the predictive capacity of a standard model based solely on static measures[42]. A number of recent studies have also attempted to predict extubation outcome using various recurrent neural networks architectures, with varying degrees of success [43-45]. It has also been shown previously that slow recovery to baseline levels of minute ventilation following successful spontaneous breathing trials is a risk factor for failure of extubation[46,47]. Such slow recovery to baseline levels aligns with what would be observed as a result of CSD prior to critical transitions in nonlinear dynamical systems [13].

Methods

Q4. The data collection and cohort segregation process could be explained in more detail in the main text, in addition to the new Figure 1. Specifically:Provide the total number of extubations analyzed and how many failed, to give a sense of the sample size.Clarify if any exclusion criteria were applied (e.g. minimum duration of data before/after extubation).

In addition to the figure 1, the number of analysed extubations and the corresponding failures have now been mentioned in the main text. These numbers correspond to numbers prior to pre-processing steps, hence excluding data losses due to the minimum duration of 2 hours required between extubation and reintubation (detailed from lines 175 in the manuscript). The final numbers after such data losses are presented in a new Table 2 in the manuscript, and detailed further in response to Q8.

As seen in Figure 1, cohorts 1, 2, 3 and 4 have sizes of 184, 2479, 333 and 38 respectively, prior to pre-processing. For patients who did not die in care, this corresponds to a failure rate of about 6.9 %.
Moreover, since the calculation of the quantifiers use windows of 60 minutes of data (see section 2.5), any ICU visit where the duration between the failed extubation and critical point (either re-intubation or death) is less than 120 minutes was removed. This is so that the window size is not greater than half of the length of the time series, which would fail to pick up nuances in the data and provide inaccurate estimations.

Q5. The statistical methods section could be expanded to:
Define the CSD metrics (autocorrelation, variance) more precisely and explain how they are calculated from the vital sign time series.

The CSD metrics are now explained with equations, detailing how the quantifiers are calculated for each window.

For a time series X = {x1, x2, ..., xn}, a window of size m and starting time t, would be given as X{t,m} = {xt, xt+1, ..., xt+m}. The variance and autocorrelation of each window would then be given by

$$\sigma^{2,t} = \frac{\sum_{i=t}^{t+m}(x_i - \bar{x})^2}{(m-1)}$$

$$r_1^t = \frac{\sum_{i=t}^{t+m-1}(x_i - \bar{x})(x_{i+1} - \bar{x})}{\sum_{i=t}^{t+m}(x_i - \bar{x})^2}$$

The resulting σ2,t and rt1 form a secondary time series, as the start point of the window,t, is slid forward, which shows how these measures change over time

Q6. Justify the choice of a 5% significance threshold for the CSD metric increases.

This exploration is the first that explores CSD in the context of extubation outcome. Therefore we could not estimate a significance threshold based on expected effect sizes. Hence the commonly used threshold of 5% ($\alpha = 0.05$) was used for detecting increases in the CSD metric. Since multiple tests are being conducted, we also used a Holm-Bonferroni approach to correct for these.

Since the application of CSD to detect the need for reintubation is a novel approach, it is important to minimize the risk of missing clinically relevant changes. Hence the most commonly used α level of 0.05 was chosen over a stricter threshold, while also correcting for multiple testing using the Holm-Bonferroni approach.

Q7. Describe the logistic regression model in more detail - what were the predictor variables and how were the magnitudes of CSD metric increases incorporated?

The full logistic regression models used are now listed in the main text, with details of the results including the log of the odds, standard error, z-values, p-values, degrees of freedom, AIC and R2 given in Table 6.

The logistic regression models used in the analysis are defined as follows.

1. $\text{ExtOutcome} \sim HR^{\tau}_{r_1} + HR^{\tau}_{\sigma 2}$
2. $\text{ExtOutcome} \sim RR^{\tau}_{r_1} + RR^{\tau}_{\sigma 2}$
3. $\text{ExtOutcome} \sim ABP^{\tau}_{r_1} + ABP^{\tau}_{\sigma 2}$
4. $\text{ExtOutcome} \sim HR^{\tau}_{r_1} + HR^{\tau}_{\sigma 2} + RR^{\tau}_{r_1} + RR^{\tau}_{\sigma 2} + ABP^{\tau}_{r_1} + ABP^{\tau}_{\sigma 2}$
5. $\text{ExtOutcome} \sim HR_{mean} + RR_{mean} + ABP_{mean}$
6. $\text{ExtOutcome} \sim \text{All Variables}$

Here $Variable_{r1}^{\tau}$ and $Variable_{\sigma 2}^{\tau}$ represent the magnitude of the Kendall correlation coefficient for the autocorrelation and variance for each variable (HR,RR and ABP). The full results of the logistic regression models, including the logs of the odds (estimate), standard errors, z-values, p-values, degrees of freedom (N), Akaike information criterion (AIC) and R2 values are summarised in Table 6.

Results

Q8. Report the total number of extubations analyzed and failure rate, to provide context for the findings.

The results section is now introduced with the final numbers of analysed files for each cohort and the corresponding extubation failure rate.

In the following sections, we present the results of the statistical analyses conducted ,including z-tests for significant increases in EWS, t-tests for comparing the magnitudes of increase, and a series of exploratory analyses to further investigate the trends in the data. The final number of files analyzed per ICU ward, cohort, and variable is shown in Table 2. This results in failure rates ranging from 5.4% to 6.4%, depending on the variable used without subdivision into wards, and from 3.9% to 14.8% when considering wards separately.

Q9. Providing the mean and standard deviation of the CSD metric increases for the success and failure groups, not just the p-values and effect sizes.

Table 4 is now modified to include the means and standard errors of the Kendall tau correlation coefficients for the success and failure groups, in addition to the p-values.

The means, standard errors and p-values corresponding to each of these tests are shown in Table 4 with the significant results highlighted.

Q10. Reporting the full logistic regression model results (coefficients, p-values, odds ratios) rather than just the model comparison.

The full results of the logistic regression model including the estimates (log of the odds ratio), standard errors, z-values, p-values, degrees of freedom, AIC and R2 values which were listed in Table 5 are highlighted further in the main text. In addition, the values for the intercept for each of the models which were previously missing have now been added to Table 5. In addition, some errors in the final estimates of the results in the previous version have been corrected.

The full results of the logistic regression models, including the logs of the odds (estimate), standard errors, z-values, p-values, degrees of freedom (N), Akaike information criterion (AIC) and R2 values are summarised in Table 6.

Discussion

Q11. Acknowledge the limitations of the study, such as the small sample size and single-center nature of the data.

We thank the editor for pointing out these important limitations of the study. We have now highlighted these in the discussion section of the manuscript.

Although the small sampling time of the vital signs time series is rare in similar studies on extubation failure, the sample size of the data is comparatively small, especially in comparison to the MIMIC datasets which have been used extensively to study extubation failure [71,72].

Furthermore, we use only data from a single paediatric hospital in London, and our results may not directly translate to an adult population.

Q12. Discuss potential confounding factors that could influence the vital sign dynamics, such as medications, fluid status, etc.

Potential confounding factors such as medication and fluid status have now been mentioned in the discussion section, with appropriate references.

Moreover, a number of potential confounding factors could influence the dynamics of the vital signs considered in this work. These include influences from drug based interventions which could alter heart rate and blood pressure, as well as the patient's fluid balance which relates to respiratory and cardiovascular stability[66,67].

Q13. Speculate on how CSD metrics could potentially be used clinically in the future (e.g. as part of a predictive model for extubation failure) while acknowledging the current limitations.

The concluding lines have been expanded to include speculation of how CSD metrics could be used in clinical practice in future, while mentioning the limitations pointed out in our results.

While our results indicate that CSD metrics on their own may not be a reliable predictor of extubation outcome, they could constitute novel features in machine learning based prediction models and enhance their predictive power [42]. This would be a promising direction for future research to potentially use CSD metrics in clinical practice, which would in turn be a significant stride towards using dynamical systems theory for prediction in the ICU.

We thank the editor again for the insightful comments on the manuscript. We hope that with these changes we have largely addressed their concerns and the manuscript can now be considered for publication in PLoS One.
