# Supplementary material for: Transitions in intensive care: Investigating critical slowing down post extubation
Source: PLoS One. 2025 Jan 24;20(1):e0317211. doi: 10.1371/journal.pone.0317211 (PMC11760018; doi:10.1371/journal.pone.0317211)
Supplement: Attachment — Submitted filename: SVG_ Response Letter_PONE-D-24-07937R2.pdf [file pone.0317211.s008.pdf]

PONE-D-24-07937R2: Response to Referees

We thank the referee for their questions and comments. Modifications made in response to these have improved the manuscript considerably, and we hope that the referee is satisfied with the revisions made. Queries are marked in bold, and our responses are provided in italics.

Reviewer #4: This study concerns an investigation of the presence of critical slowing down (CSD) prior to failed and successful extubation events in pediatric patients. The presence of CSD as signaled by rising autocorrelations and variances was examined in several physiological variables. Results showed very limited predictive promise, and the authors consequently conclude that this method is not clinically useful yet. On the whole, this investigation is well-written and despite the modest findings, it is a worthwhile addition to the growing literature on CSD-based early warning signals in medicine.
I was happy to see that the authors included cases in which CSD was not expected so that they can give a better understanding of the presence of EWS by calculating predictive values. Moreover, I appreciated the use of a single-case analysis to inform and preregister the methods of the current study.

We thank the reviewer for their accurate summary and their comments on the findings. As the reviewer points out, our work was pre-registered in an effort to limit biases that could arise as a result of extensive explorations in the dataset. Although the original single-case analysis showed promise, the final analysis on the entire sample indicated that CSD is rare prior to re-intubation.

After reading the manuscript, I have several comments that deserve to be addressed prior to publication. I will begin with the more major comments and list minor comments below.

In line with the reviewers recommendations, we have now significantly changed the results and discussion section to address many of the points raised. New subsections have been added to the discussion section to improve clarity and accessibility for readers. Many of the answers and changes made to the manuscript feed into multiple questions raised by the reviewer. For instance, improving accurate identification of transition points may lead to higher performance in future studies. In such instances the answers are only mentioned in response to the questions where they are most relevant for the sake of brevity. Changes to the manuscript are mentioned in response to the queries in red, with grey lines indicating sections from the previous version of the manuscript which are added for clarity.

Major comments:

1. Like many other studies on CSD in medicine to date, this study's results do not yet provide much encouragement to pursue these methods in the context of clinical predictions. While combinations of variables and additions to machine learning models may yet improve the predictive abilities, much remains unknown about how to identify transition points, and how best to calculate CSD (relevant time scale, required data length and density, window size in moving-window analyses, AR/SD vs flickering, etc.). While the authors do note these difficulties in their discussion, I would like to see them make more concrete recommendations for future research.

We thank the reviewer for this comment. As the reviewer rightly points out, this study aligns with many other investigations on CSD in medicine, most of which show limited potential for clinical prediction. The reviewer points out a number of difficulties in identifying transition points and calculating CSD in data. Each of these points are indeed relevant to the discussion and have now been added in the new version of the manuscript. Subsections have now been added, expanding on the previous version of the manuscript, on 4.3 Reasons for low model performance, 4.4 Limitations and 4.5 Future directions. Specifics are discussed in response to the sub points raised below

1.1. For instance, the authors comment on several inherent limitations in the accurate categorization of true and false positives in the discussion (lines 420-437). Their relatively concrete outcome categorization is a strength in this study, even if it may still be somewhat subjective. I would like to see a more fleshed-out discussion of how these problems might be addressed in future studies. How do they envision that transition points can be more accurately identified? And in a real-time setting, at that.

We thank the reviewer for this question. The categorization of extubation failures and determination of exact extubation times is indeed a strength of this study. Despite this, a number of systematic issues remain in the objective identification of transition points in this study. Some of these issues were pointed out in the previous version of the discussion. New additions to the discussion section are now shown below.

Section 4.4 Limitations

As mentioned above, a major limitation in studies on extubation is in identifying true physiological transition points. In clinical practice, the decision to re-intubate is made preemptively in anticipation of potential instability, rather than waiting for definitive evidence of a transition. As a result transition points in this study may not indicate a change in the dynamics of the system, unlike in other fields where EWS have been detected. This study uses the timing of re-intubation as an approximate transition point, which may often be far from the true transition point.

Section 4.5 Future directions
To overcome the limitation of the subjectivity associated with transition points, future studies could focus on instances where emergency reintubation was required. In such cases, the actual point of reintubation may be much closer to the transition point than in a general sample. Another possibility could be to quantify markers of instability, such as deviations in physiological baselines using methods such as change point analysis. Only re-intubations that also include significant quantitative change would then be checked for early warning signals, as done in studies on CSD in other clinical contexts [85].

1.2. Likewise, much remains unknown about the optimal way to analyze time series data to identify CSD. Do the authors have any suggestions on how to systematically examine the hurdles they list (and I listed above)?

Many methodological papers have considered how to analyse time series data for identifying CSD either in general or specific contexts. Based on the literature and our findings, general guidelines for detrending, accounting for serial correlations, and choosing window sizes may be effective but require context-specific adjustments. Many of the choices in this paper were made in line with literature and tested out in a single case. The null results of this study suggest that the issues identified in the single individual may not generalize to the whole dataset. Perhaps future studies would benefit from using a small subsample instead of a single dataset to determine the best parameters for detecting CSD in the entire data. Additional considerations for analysing time series data to identify CSD have been listed in section 4.3 and mentioned in response to point 3.3.

4. Discussion
4.3 Reasons for low model performance
In our study we attempted to overcome these difficulties by systematically examining and overcoming them in a single test case [48]. However the largely negative results of our study on the entire dataset seems to suggest that either the issues identified on the single dataset did not generalize to the whole system. To avoid this, future studies in such complex biological datasets may benefit from using a small, representative subset (perhaps 5%) of the dataset to optimize parameters for identifying CSD.

1.3. Moreover, while their group-level combined logistic regression model shows somewhat better outcome prediction, this is not all that useful at an individual level. Can the authors translate this part of their study to avenues for future studies? For instance, do they recommend that studies combine multiple variables and signals to improve their predictive performance at single-case level? Or should the group-level results inform a different direction of study (e.g., machine-learning models only).

The reviewer correctly points out that the results of the logistic regression model is a group level analysis which does not directly translate to the level of the individual. One way to translate this analysis to an individual level is to calculate the mean and CSD variables over a sliding window and calculate a combined metric that predicts extubation outcome. And as already mentioned in the previous iteration of the manuscript, CSD metrics could also be used as a feature to train machine learning models, which could then be used to predict need for reintubation in new unseen samples. These have now been added in the recommendations for future research.

4. Discussion
4.5 Future Directions
Adding CSD metrics to the mean levels of the variables also showed a significant improvement over the model without them. At an individual level this could indicate that the mean and CSD metrics calculated over a sliding window could predict extubation outcome better than using either of them alone. Going even further, while CSD metrics on their own may not be a reliable predictor of extubation outcome, they could constitute novel features in machine learning based prediction models and enhance their predictive power[42].

1.3.1. Have they considered using combinations of variables at single-case level for their own study?

While this was not considered before, we have now done two additional analyses at the group level to check whether this would yield better results. The first tested how the sum of strengths of positive transitions (measured as the sum of positive τ values) differed between Cohorts 1 and 2. We saw that Cohort 1 had a significantly higher value for this than Cohort 2 in a one sided t-test. The AUC calculated by varying this metric gave a value of 0.597, higher than any of the individual metrics. We have now added these to the results section and discussed the implications for future studies in the subsection on future directions.

3. Results
3.4 Model performance
Since model 4 that combines the HR,RR and ABP variables performed better than models 1,2 and 3 individually, a one-sided t-test was conducted to check if Cohort 1 had a higher strength of positive transitions across variables (defined as the sum of positive τ or all variables) as compared to Cohort 2, yielding significant results (t-statistic=2.056, p-value=0.022). Moreover, the AUC of the combined strength of positive transitions was 0.597, higher than the AUC for the individual variables.
4. Discussion
4.4 Future Directions
A significant outcome of the present study is the improvement seen by using CSD variables in a simple logistic regression model. At the group level, the combination of CSD variables showed lower AIC and higher R^2 than models using individual CSD variables. This difference persisted even when the degrees of freedom were kept the same. This indicated that a combination of variables could perform better than individual CSD variables to detect extubations. Moreover Cohort 1 showed a higher strength of positive transitions across variables as compared to

Cohort 2, and a higher predictive capacity (measured using AUC) as compared to the individual variables. This indicates that using a combination of EWS at an individual level could reduce misdetection and increase sensitivity in the sample.

2. I expected to see some discussion of the distribution of tau values they found. A stronger positive tau value over the autocorrelation and variance time series is taken to be a sign of CSD. However, in Figure 4 we also see that many negative trends in tau were found (in both C1 and C2). What might explain this? Are there theoretical or practical factors (data quality, time scale?) that could account for this?

I am aware of at least one more theoretical article that shows that declining trends in the variance may occur in particularly noise or insufficient data (Dakos, Van Nes et al., 2012 doi.org/10.1890/11-0889.1). The autocorrelation is expected to be more robust to this.

The negative trends observed in Figure 4 are indeed quite interesting. Several factors, including noise or insufficient data, as noted by the reviewer, could contribute to such trends. Theoretical causes could include flickering prior to a transition or processes that exhibit critical speeding up. In complex biological data such as ours a combination of factors could be at play leading to the large number of negative trends observed in data. We have now included a paragraph discussing the distributions of tau values in Figure 4.

4. Discussion

4.1 Summary and significance

One of the interesting features of the study is the distributions of the Kendall τ coefficients in Figure 4. Apart from strong false positives and negatives, we also see a number of negative trends in both cohorts 1 and 2. There is some evidence that such negative trends could occur in autocorrelation in systems exhibiting flickering prior to transitions or in both autocorrelation and variance as a consequence of processes that exhibit critical speeding up [65 –67]. The variance is also known to decrease when there is insufficient data and the fluctuations in the system are dominated by low frequencies or when the data is highly noisy [68]. In complex biological data, such as this, both issues related to data, as well as competing processes with opposite effects could lead to such negative trends.

3. On line 336-338, the authors discuss the sensitivity and specificity values they found and suggest that in the few cases (~20%) that the model gives a warning signal, the ~80% specificity means that those cases might actually be mostly correct. I do not believe this is an entirely correct interpretation.
The specificity or true negative rate indicates the proportion of cases in which a true negative is correctly identified (thus, Cohort 2 is classified as not having an EWS), and does not directly relate to the correctness of positively flagged cases (Cohort 1). In fact, since ~20% of Cohort 2 is incorrectly flagged as a positive (the false negative rate, 1-Specificity) and the sample is so much larger, the chances of a positively flagged test actually being a true positive are incredibly small, around 7% (as seen in the PPV). To put

it very clearly, the false discovery rate is nearly 94% (1-PPV) across their measures, so the interpretation they have given is not supported.
My recommendation to the authors is threefold:
3.1. Rewrite the interpretation on line 336-338 to more accurately reflect their findings, they are very modest at best.

We thank the reviewer for pointing out this error. As they rightly point out, the inflated specificity is indeed simply due to the class imbalance in the problem, and not suggestive of the correctness of the positive class. The PPV correctly shows this, and the previous incorrect interpretation is now removed. The previous sentence interpreting the high specificity as the proportion of correctly identified cases is now removed and replaced with the following.

3. Results
3.4 Model performance
The low sensitivity and PPV values indicate that the model has limited predictive power... Since a significant proportion of the much larger Cohort 2 is incorrectly flagged as a positive, the chances of a positively flagged test actually being a true positive are incredibly small, as seen in the PPV.

3.2. Use a metric of the accuracy that accounts for unbalanced samples. For instance, the Balanced Accuracy ((Sensitivity + Specificity)/2) does not rely on the group sizes. The F1-score is another option, as it gives a higher weight to finding true positives, though it does still rely on group ratios (as per their own note about PPV/NPV on line 340).
3.2.1. Out of curiosity I calculated both those metrics from the confusion values in Table 5, and the Balanced Accuracy was around .5 (chance-level in a balanced dataset) across their measures, while the F1 scores were around .1. Both metrics range from 0 to 1, with 1 being optimal prediction, so a restrained conclusion about their findings is called for.

As suggested by the reviewer, two new metrics that account for class imbalance are now added to Table 5, namely the balanced accuracy and the AUC of the ROC curve. The ROC curve was generated by varying the threshold of the τ metric, while avoiding negative τ values (setting these to a minimum negative value). Both metrics show limited predictive value, as pointed out by the referee, close to the chance prediction of 0.5. The autocorrelation of the heart rate and the variance of the blood pressure show the highest performance in these two quantifiers, in alignment with the other results.

3. Results
3.4 Model performance
The metrics used to evaluate the predictive capabilities of the model are Positive Predictive Value (PPV), Negative Predictive Value (NPV), Sensitivity, Specificity, Balanced Accurancy (Acc_B) and Area Under the Receiver Operator Characteristics (ROC) Curve (AUC). All quantifiers have values that vary between 0 and 1, with 1 indicating ideal classification performance. The PPV, NPV, Sensitivity and Specificity are affected by class imbalance,

whereas Acc_B and AUC are robust to this. Values close to 0.5 for the latter indicate classification no better than chance.

In this study, since we are interested in only predictions due to positive trends, the ROC curve was generated by varying the threshold of the Kendall τ coefficient, while setting negative τ to a minimum negative value (effectively excluding them).

The values close to 0.5 of Acc_B and AUC indicate that the model has limited predictive power.

3.3. To more fairly represent their findings, and to tie in with the clinical nature of their data, a discussion of the number of false negatives (missed signals) is also warranted. Missing three quarters of the cases one would actually want to identify to prevent extubation failure, is obviously quite problematic. The authors could discuss how they might work on improving the sensitivity (changes to their analytical methods?), even if at the cost of specificity.

The reviewer is right in pointing out that in clinical contexts missing transitions is likely to have much higher consequences than having a specific signal. We suggest a change in the methodology in the lines of one of the suggestions of the reviewer, combining EWS for a better prediction. We notice that the AUC of the total strength of positive signals is higher than any of the individual EWS.

In addition we also suggest using the chance of transition quantified through a suitable metric could aid in decision making in the ICU. By replacing binary outcomes with a probability, the decision to reintubate could be taken by the clinician while taking into consideration other clinical cues.

4. Discussion

4.3 Reasons for low model performance

In the context of critical care, missed detections are more crucial than false detections, since the former can prevent timely interventions. Hence it may be more important for future studies to improve sensitivity, even at the cost of specificity.

4.5 Future directions

Setting an optimal threshold for this combined strength of positive transitions could increase sensitivity even at the cost of specificity, which may be desirable in clinical contexts.

…

In a real time setting, binary outcomes could be replaced with a continuous measure that gives the probability of failure, which could aid in clinical decision support [86]. In the context of the CSD, this could be the Kendall τ values, or the deviations from baseline for a sliding window mean, or prediction probabilities for a machine learning algorithm.

Please elaborate further on the low specificity and missed signals in the discussion, alongside further interpretation of any new metrics that might be added in response to my previous point.

Both low specificity and sensitivity can occur in data due a number of theoretical and practical considerations. The former could be due to either no bifurcation in the system or due to the variables considered not being aligned with the direction of the transition. Competing processes (such as critical speeding up) could also mask transitions in the data. Practical considerations such as time scale of interest, under sampling, trends in data etc could also contribute to false positives and negatives. These issues are now elaborated in the new version of the manuscript. New additions to the discussion are highlighted below.

4. Discussion

4.3 Reasons for low model performance

Although increasing variance and autocorrelation have analytically been proven to be signals of CSD, these have often been difficult to observe in complex biological systems [69, 73, 74]. Several factors may contribute to this. For instance, the system itself may not be undergoing a bifurcation that is expected to be theoretically preceded by CSD [75]or the variables under consideration may not be in the direction of slowest recovery, where EWS are most prominent [76– 78]. Moreover, competing processes could lead to critical speeding up of the dynamics or flickering of the transition which could mask detection of EWS in the system [65, 67, 69].

Beyond these theoretical considerations, practical issues such as under-sampling, insufficient data and time scale of occurrence could lead to false negatives in real time series [79– 81]. False positives on the other hand can occur due to serial correlations and time dependent trends [56, 61]. While detrending can remove long term trends, too small a bandwidth removes important trends, and increases false negatives whereas too long a bandwidth may not remove all irrelevant trends and consequently increase false positives [82]. It has been suggested that in systems where naturally strong fluctuations exist in the system response, other early warning signals that quantify flickering could be more useful in detecting transitions [13, 66]. Metrics derived from detrended fluctuation analysis and significance testing using bootstrapping methods have both shown potential as robust approaches for detecting CSD in real-world scenarios [4,12,58]

In our study we attempted to overcome these difficulties by systematically examining and overcoming them in a single test case [48]. However the largely negative results of our study on the entire dataset seems to suggest that either the issues identified on the single dataset did not generalize to the whole system.

4. Overall, I recommend that the authors are more circumspect about their findings and how much promise they hold. While the discussion has not been written to give overly optimistic interpretations or inflated importance to their results, it can be stated more clearly that their findings are mostly null, with very poor predictive values. Perhaps the possibility that using CSD to predict transitions in medicine is NOT a worthwhile avenue to investigate further should be mentioned in light of their, and other studies' limited findings. As mentioned above, and by the authors themselves, studying CSD in medicine is difficult, and centering their discussion more around the prerequisite advancements in theoretical and methodological understanding of biological systems might be fitting.

We agree that future investigations of CSD in biological systems should be more measured in their expectations, in light of the difficulties of such studies in complex biological data. Perhaps focussing on CSD based metrics as one of many features of a transition in a machine learning model, or combining EWS from different variables or with other metrics could be a possible avenue for future investigations. We have concluded the discussion section with a paragraph that is more circumspect about the findings of the study and possible directions for future studies, in line with the suggestions of the reviewer

4.Discussion
4.6 Conclusions
Predicting extubation outcome is an important question that has been studied extensively in the past, with mixed results. The results of the present study found limited evidence to support the use of CSD based metrics to predict extubation outcome, with only the autocorrelation of the heart rate showing significant differences between the groups. These results highlight the challenges of detecting critical slowing down in complex biological systems in general, and more so in critical care. The findings point towards the need for a marked change in the way CSD based metrics are currently used for prediction in the context of medicine. For instance, combining CSD based metrics along with other indicators of extubation failure, or using them as one of many features in a machine learning algorithm could lead to improved prediction. This could perhaps be the way for future researchers looking to use CSD metrics or other measures from dynamical systems theory for prediction in the ICU.

Minor notes:

On line 87, the term EWS is first introduced, but it is never written out in full. It may also be worth briefly explaining the relation between CSD and EWS for readers who are less embedded in this topic.

The term EWS is now introduced earlier in the manuscript and the relationship between CSD and EWS are now mentioned in the first paragraph of the introduction

These metrics which characterize this increased relaxation time due to CSD are called early warning signals (EWS).

Page 6, Figure 1: the flowchart includes the same outcome twice "Monitor data between extubation and pseudo-reintubation times". Is that correct? The note mentions for Cohort 1 and 3 "monitor data … between extubation and re-intubation" – so without pseudo. Please make sure the figure is correct.

This was a typo. We thank the reviewer for pointing this out. This is now changed in the revised version

Typo on line 212: the word 'variance' here should be 'autocorrelation', the topic of the hypothesis.

This is now fixed in the revised version

For Table 3, is the title as intended? It currently reads "The proportions of significant Mann-Kendall hypothesis tests conducted on cohort 1" but it reports both C1 and C2 and the different variables and EWS.

This is now changed in the revised version

Line 283: the authors write "a greater value of tau", the authors could consider specifying that a greater positive value of tau is what is expected. It may be clear from the tested hypotheses that follow shortly after, but it cannot hurt to use precise language here. That would also help set up a later discussion of their results, as I have suggested above in comment 2.

This is now changed in the revised version

Table 5, similar issue to Table 5 in that the table is described as referring to Cohort 1 only (and includes this label in the first column header). However, the authors are reporting true and false positives and negatives. The FP and TN values are based on cohort 2, so I recommend that the authors update their labels.

This is now changed in the revised version

We once again sincerely thank the reviewer for their insightful comments which have considerably improved the manuscript. We hope we have fully addressed the reviewer's comments.
